# Dr. R. Stansbury Sutton

**Published:** 1906-06

**Authors:** 


Dr. R. Stansbury Sutton, of Pittsburg, a graduate of the University of Pennsylvania, 1865, died suddenly from heart disease in a street car in the city of his home, April 21,1906, aged 65 years. He was a prominent gynecologist and a member of various societies, city, state, national, and foreign, and served as Chief Surgeon of brigade in the war with Spain. He was a ready speaker and courted controversy on the floor. He was surgeon of a private hospital for women which he conducted for many years.

